# Enhancing Toughness and Reducing Volumetric Shrinkage for Bis-GMA/TEGDMA Resin Systems by Using Hyperbranched Thiol Oligomer HMDI-6SH

**DOI:** 10.3390/ma14112817

**Published:** 2021-05-25

**Authors:** Biao Yu, Jingwei He, Sufyan Garoushi, Pekka K. Vallittu, Lippo Lassila

**Affiliations:** 1 Department of Biomaterials Science and Turku Clinical Biomaterials Center-TCBC, Institute of Dentistry, University of Turku, 20520 Turku, Finland; y.biao@lingnan.edu.cn (B.Y.); msjwhe@scut.edu.cn (J.H.); sufgar@utu.fi (S.G.); pekval@utu.fi (P.K.V.); 2 School of Chemistry and Chemical Engineering, Lingnan Normal University, Zhanjiang 524048, China; 3 College of Materials Science and Engineering, South China University of Technology, Guangzhou 510642, China

**Keywords:** toughness, volumetric shrinkage, hyperbranched thiol oligomer

## Abstract

In order to improve the toughness and reduce polymerization shrinkage of traditional bisphenol A-glycidyl methacrylate (Bis-GMA)/triethylene glycol dimethacrylate (TEGDMA) based dental resin system, a hyperbranched thiol oligomer (HMDI-6SH) was synthesized via thiol-isocyanate click reaction using pentaerythritol tetra(3-mercaptopropionate (PETA) and dicyclohexylmethane 4,4′-diisocyanate (HMDI) as raw materials. Then HMDI-6SH was mixed with 1,3,5-Triallyl-1,3,5-Triazine-2,4,6(1H,3H,5H)-Trione (TTT) to prepare thiol-ene monomer systems, which were added into Bis-GMA/TEGDMA resins with different mass ratio from 10 wt% to 40 wt% to serve as anti-shrinking and toughening agent. The physicochemical properties of these thiol-ene-methacrylate ternary resins including functional groups conversion, volumetric shrinkage, flexural properties, water sorption, and water solubility were evaluated. The results showed that the incorporation of HMDI/TTT monomer systems into Bis-GMA/TEGDMA based resin could improve C=C double bond conversion from 62.1% to 82.8% and reduced volumetric shrinkage from 8.53% to 4.92%. When the mass fraction of HMDI/TTT monomer systems in the resins was no more than 20 wt%, the flexural strength of the resin was higher or comparable to Bis-GMA/TEGDMA based resins (*p* > 0.05). The toughness (it was measured from the stress–strain curves of three-point bending test) of the resins was improved. Water sorption and water solubility tests showed that the hydrophobicity of resin was enhanced with increasing the content of thioester moiety in resin.

## 1. Introduction

Dental resin composites that based on photosensitive dimethacrylate monomers, such as bisphenol A-glycidyl methacrylate (Bis-GMA), triethylene glycol dimethacrylate (TEGDMA), and urethane dimethacrylate (UDMA), have been used as dental restorative materials for several decades due to their sufficient mechanical properties, acceptable aesthetics properties, and good bond ability to tooth tissue. Unfortunately, volumetric shrinkage and polymerization shrinkage stress of resin composites lead to restorations marginal microleakage and secondary caries, which reduce the clinical performance of these materials [1,2]. During the photopolymerization process, the distance between monomers is reduced from Van der Waal force (~4 Å) to covalent bond distance (~1.5 Å), and the free volume is reduced too. Simultaneously, shrinkage stress occurs when the contraction is obstructed and the material is rigid enough to resist sufficient plastic flow to compensate for the original volume [3]. The factors involved in the development of stress in dental resin composites are complicated. It was found that inorganic fillers, resin matrix, coupling agent, and polymerization process would affect the polymerization shrinkage stress [4].

Among these factors, the resin matrix is considered one of the most important factors [3]. The researchers have developed many strategies to reduce volumetric shrinkage and shrinkage stress. For instance, increasing the content of fillers is an easy strategy to reduced volumetric shrinkage and shrinkage stress [5]. The principle of this strategy is that the higher the content of filler, the lower content of the resin matrix prone to shrinkage. However, the flowability of dental resin composites would be reduced too [6]. Similarly, the increasing molecular weight of monomers to reduce the number of photo-activated groups per molecular is a useful strategy to reduce volumetric shrinkage and shrinkage stress. Still, the viscosity of these monomers would also be increased with high molecular we1ight [7].

Ring-open monomers such as epoxy-functionalized siloxanes [8], spiro orthocarbonates [9,10], cyclic ketene acetals [11], and vinylcyclopropanes [12,13] are usually used as anti-shrinkage agents in dental resins to reduce volumetric shrinkage and shrinkage stress. The mechanism is that they can undergo cation or radical ring open polymerization wherein bonds are broken for each new bond formed. The broken bonds can result in volume expansion to counter the volume contraction when the new bond formed [14].

Different from dimethacrylate based resins, the polymerization of thiol-ene based resins is the step-growth mechanism, resulting in more homogeneous polymeric network formation and high monomer conversion, delayed gel point, and lower polymerization shrinkage and shrinkage stress [15]. Bowman’s group firstly reported thiol-ene resins used as dental restorative materials in 2005 [16]. They found that the oligomers of thiol-ene resins had lower volumetric shrinkage and shrinkage stress than Bis-GMA/TEGDMA resin systems, while mechanical properties and glass transition temperature needed to be improved. Subsequently, they incorporated thiol-ene mixture as diluents into conventional dimethacrylate resins to prepare thiol-ene-methacryalte ternary resins. It was found that shrinkage stress reduction while maintaining equivalent flexural modulus [17,18]. They also prepared that ester-free thiol-ene dental restorative materials, and found that the polymerization shrinkage stress and water uptake were dramatically reduced, and the toughness of materials was increased [14,19]. He et al. [20] synthesized a novel fluorinated allyl ether and mixed it with pentaerythritol tetra (3-mercaptopropionate) (PETMA) at a molar ratio of 1:1 to form a new thiol-ene resin system. They incorporated these thiol-ene monomer systems into Bis-GMA/TEGDMA resins and found that volumetric shrinkage and shrinkage stress, as well as water sorption and solubility, reduced.

Hyperbranched and dendritic molecules are special topology structure molecules with a central core, a high molecular weight, and many end groups. They have lower viscosity with similar molecular weight compared to their linear analogous counterpart [21]. It is beneficial to reduce volumetric shrinkage and shrinkage stress [22,23] by adding these monomers into dental resins to reduce the content of C=C double bonds in resin without increasing viscosity. Some researchers have reported that the incorporation of methacrylate functional end groups hyperbranched or dendritic oligomers into dimethacrylate resins systems to reduce polymerization shrinkage. In our previous study, we have synthesized a dendritic methacrylate oligomer G-IEMA [24]. By adding this dendritic oligomer into UDMA/TEGDMA resin systems, the volumetric shrinkage was reduced with the increasing G-IEMA content in the resin. However, the synthesis process of these dendritic monomers is always tedious and expensive.

Herein, a hyperbranched thiol oligomer was synthesized by thiol-isocyanate click reaction and incorporated into Bis-GMA/TEGDMA based resin with the aim of reducing volumetric shrinkage due to thiol-ene step-growth mechanism and reducing methacrylate group concentrate on in the resin. The physicochemical properties such as mechanical properties, monomer conversion, water solubility, and water sorption were also investigated.

## 2. Materials and Methods

### 2.1. Materials

2,2-bis [4-(2-hydroxy-3-methacryloyloxypropoxy)phenyl]propane (Bis-GMA), triethylene glycol dimethacrylate (TEGDMA), and Pentaerythritol tetra(3-mercaptopropionate) (PETMP) were purchased from Sigma-Aldrich (Shanghai) Trading Co., Ltd. Shanghai, China. Dicyclohexylmethane 4,4′-diisocyanate (HMDI), Phloroglucinol (TPX) were obtained from Beijing InnoChem Science & Technology Co., Ltd. Beijing, China. 1,3,5-Triallyl-1,3,5-Triazine-2,4,6(1H,3H,5H)-Trione (TTT), Camphorquinone (CQ), Triethylamine (Et_3_N) and 2-(Dimethylamino)ethyl methacrylate (DMAEMA) were purchased from Shanghai Titan Scientific Co., Ltd. Shanghai, China. All chemicals were used without further purification.

### 2.2. Synthesis of Hyperbranched Thiol Oligomer HMDI-6SH

**Figure 1:**
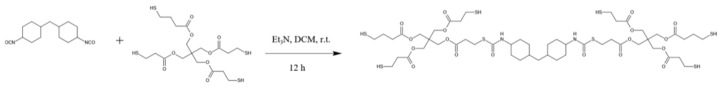
Synthesis route of hyperbranched thiol oligomer HMDI-6SH.

The synthesis route of hyperbranched thiol oligomer HMDI-6SH was shown in Figure 1. 400 mL of Dichloromethane (DCM) was added into a 1000 mL three-necked round-bottomed flask equipped with a teflon-coated magnetic stir bar, and then PETA (76.64 g, 152.47 mmol, 2.00 equiv.) and Et_3_N (0.077 g, 0.76 mmol, 0.01 equiv.) were added into the flask and dissolved in DCM under nitrogen atmosphere. Afterwards, HMDI (20.00 g, 76.23 mmol, 1.00 equiv.) dissolved in 20 mL of DCM was adding into the mixture dropwise for 30 min. The reaction mixture was stirred at room temperature and monitored by FT-IR spectrum. It was completed until the peak of -NCO groups at 2230 cm^−1^ disappeared. After completing the reaction, DCM was removed on a rotary evaporator under vacuum, and the crude product was precipitated using petroleum ether for three times to remove Et_3_N. After removing the solvent under vacuum, the product was obtained as a colourless oil with a yield of 95%. The chemical structure of the thiol oligomer was characterized by FT-IR spectrophotometer ((Nicolet 6700, Thermo Fisher Scientific, Madison, WI, USA, KBr, film) and proton nuclear magnetic resonance (^1^H NMR) (AVANCE 400 MHz, Bruker, Berlin, Germany, CDCl_3_, 400 MHz). The average number of molecular weights (Mn) and the polydispersity of index (PDI) was measured by gel permeation chromatography (GPC) (PL-GPC50, Agilent, Santa Clara, CA, USA), equipped with a low-angle laser light scattering (LALLS) detector. THF was used as the mobile phase at a flow rate of 1 mL/min at 30 °C.

FT-IR (neat, KBr): 3455 cm^−1^, 3358 cm^−1^, 2927 cm^−1^, 2853 cm^−1^, 2567 cm^−1^, 1738 cm^−1^, 1671 cm^−1^, 1509 cm^−1^, 1469 cm^−1^, 1389 cm^−1^, 1354 cm^−1^, 1248 cm^−1^, 1196 cm^−1^, 1152 cm^−1^, 1051 cm^−1^, 935 cm^−1^. ^1^H NMR (400 MHz, Chloroform-d): δ 5.33 (s, 1H), 4.48–3.98 (m), 3.25–2.88 (m), 2.73–2.64 (m), 1.93–1.48 (m), 1.37–0.81 (m). Mn: 5530, PDI: 1.83.

### 2.3. Preparation of Resin Systems

**Figure 2:**
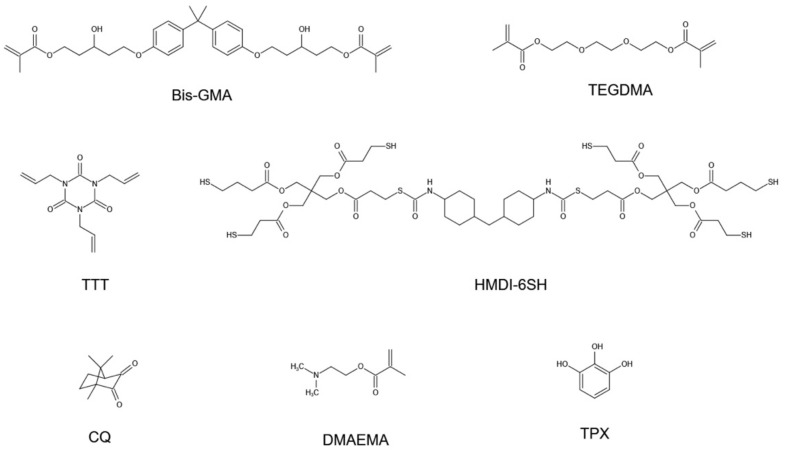
The chemical structure of monomers, photo-initiator and stabilizer used in this study.

The synthesized thiol oligomer HDMI-6SH were mixed with (1H, 3H, 5H)-Triallyl-1,3,5-Triazine-2,4,6(1H,3H,5H)-Trione (TTT) (The mole ratio of -SH groups in HMDI and C=C groups in TTT was kept at 1:1). Then, the thiol-ene mixture was added into Bis-GMA/TEGDMA resins (Bis-GMA/TEGDMA 60 wt%/40 wt%) with a series of mass ratio (10 wt%, 20 wt%, 30 wt%, and 40 wt%). The CQ and DEMEMA were served as photo-initiator and co-initiator, and their mass ratios in resin were both 0.7 wt%. The resins were marked as 10%HMDI/TTT, 20%HMDI/TTT, 30%HMDI/TTT, and 40%HMDI/TTT. Bis-GMA/TEGDMA resin without thiol-ene mixture was used as a control. The chemical structure of monomers, photo-initiator and stabilizer used in this study was shown in Figure 2. Photoactivation procedures were performed using a dental lamp (X-cure, Woodpecker, Guilin, China) with an irradiation intensity of 1000 mW/cm^2^. The uncured resins were injected into stainless steel mold (size 2 mm × 2 mm × 25 mm), which were sandwiched between two glass slides. Both sides were irradiated for 60 s to ascertain uniform conversions throughout the sample thickness. A total of 18 specimens of each model resins was made for flexural properties (n = 10), volumetric shrinkage (n = 3), and water sorption and solubility analyses (n = 5). The specimens were randomly allocated into test groups.

### 2.4. Functional Groups Conversion Measurement

The degree of functional groups conversion (DC%) during and after the photoinitiation of polymerization was monitored by Fourier transform infrared spectroscopy (FT-IR) (Nicolet 6700, Thermo Fisher Scientific, Waltham, MA, USA). Resin sample was coated on KBr Pellets to form a very thin film, and the absorbance peak of uncured samples was obtained. Then photo-polymerization of the sample was carried out by irradiation of a dental light source (1000 mW/cm^2^, X-cure, Woodpecker, Guilin, China) at room temperature. Spectra during the irradiation process were recorded every 10 s for 1 min. The DC% was calculated from the aliphatic C=C peak at 1636 cm^−1^, thiol group peak at 2530 cm^−1^ normalized against the benzene C=C double bond peak at 1608 cm^−1^, according to formula (1)

(1) DC%=AfunAbenzene0−AfunAbenzenetAfunAbenzene0

where *A_fun_* and *A_benzene_* are the absorbance peak areas of functional groups (C=C double bond groups at 1640 cm^−1^, thiol groups at 2230 cm^−1^), and benzene at 1608 cm^−1^, respectively. AfunAbenzene0 and AfunAbenzenet are the normalized absorbance of the functional groups at radiation time of 0 and *t*, respectively; *DC(t)*% is the conversion of functional groups as a function of radiation time.

### 2.5. The Determination of Glass Transition Temperature (T_g_)

The glass transition temperature (*T*_g_) of the cured resins was determined by dynamic mechanical analysis (Q800, TA, New Castle, DE, USA) using an in tensile mode. Bars of 30 × 10 × 3 mm were heated from −30 to 120 °C at 3 K/min under a nitrogen atmosphere, using a frequency of 1 Hz.

### 2.6. Flexural Properties

Ten specimens with a size of 2 mm × 2 mm × 25 mm were prepared for every resin formulation. Five specimens were kept dry until the start of testing, and the other five samples were stored in distilled water at 37 °C until the start of testing (the storage time was as long as the time for water sorption and solubility test). Three points bending test (span 20 mm) was carried out to evaluate the flexural strength (*FS*) and modulus (*FM*) according to ISO 10477:92 standard with a universal testing machine (Model Z010, Zwick GmbH & Co. KG, Ulm, Germany) at a cross-head speed of 1.00 mm/min [25]. The *FS* in MPa, *FM* in GPa, and toughness (*TS*) in KJ/m^2^ were then calculated as:

(2) $$FS=\frac{3pL}{2bh^{2}}$$

(3) $$FM=\frac{SL^{3}}{4bh^{3}}$$

(4) $$TS=\frac{A}{bh}$$

where *p* stands for load at fracture (N), *L* is the span length (20 mm), *b* and *h* are the width and thickness of the specimens in mm, respectively.

*S* is the stiffness (N/m). S = F/d, *d* is the deflection corresponding to load F at a point in the straight-line portion of the trace. The FM was also determined from the slope of the initial linear part of stress–strain curve.

*A* is the area under load-deflection cure and is the energy applied on specimens in Joules (J).

### 2.7. Volumetric Shrinkage

The volumetric shrinkage (VS%) was measured using the variation of densities before and after polymerization according to Equation (5).

(5) $$VS\%=\frac{\rho_{polymer}-\rho_{monomer}}{\rho_{polymer}}\times 100\;\%\;$$

where $\rho_{polymer}$ was the densities of cured resin and $\rho_{monomer}$ was the densities of the uncured resins. $\rho_{polymer}$ and $\rho_{monomer}$ were measured by densitometer (MDJ-300M, Xiongfa Instrument, Xiamen, China), according to Archimedes’s principle. Each resin formulation was measured for three times.

### 2.8. Water Sorption and Water Solubility

To measure water sorption (WS%) and water solubility (SL%) of cured resin, the rectangular beam specimens (size 2 mm × 2 mm × 25 mm, five specimens for each resin formulation) used as three-point bending test were prepared. The dry weight (*M*_1_) of specimens was measured by a balance (MDJ-300M, Xiongfa Instrument, Xiamen, China) with an accuracy of 0.1 mg.

Then, every specimen was immersed in 30 mL of distilled water at 37 °C. At a fixed time interval, they were removed out from the water and dried to remove excess water with absorbent paper, re-weighed, and returned to the water. Equilibrium mass (*M*_2_) was obtained until there was no significant change in mass at 30th day immersion.

The specimens were dried at 60 °C until their mass was constant, and the result was recorded as *M*_3_. Water sorption (WS%) and solubility (SL%) were then calculated using the following formula.

(6) $$WS\%=\frac{M_{2}-M_{1}}{M_{1}}\times 100\;\%\;$$

(7) $$SL\%=\frac{M_{1}-M_{3}}{M_{1}}\times 100\;\%\;$$

### 2.9. Statistical Analysis

The experiments results were analyzed using one-way analysis of variance (ANOVA). Multiple pair-wise comparisons were further conducted using Tukey’s test with a significance level of 0.05.

## 3. Result and Discussion

### 3.1. Hyperbranched Thiol Oligomer (HMDI-6SH) Synthesis

**Figure 3:**
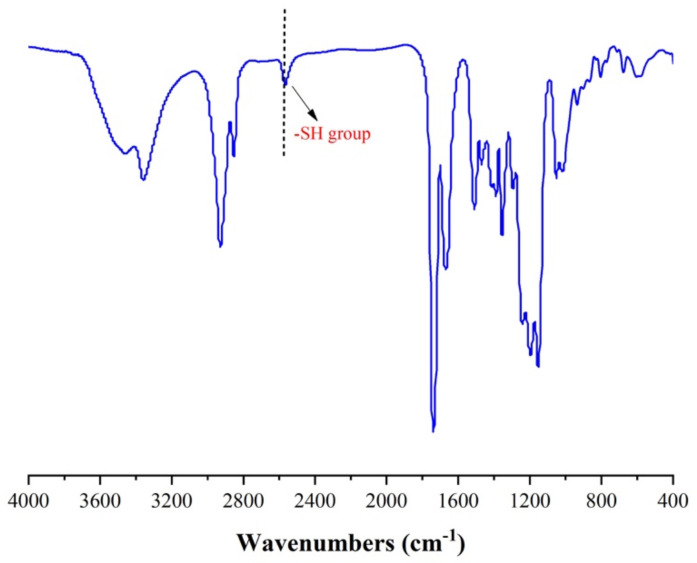
FT-IR spectrum of hyperbranched thiol oligomer HMDI-6SH.

**Figure 4:**
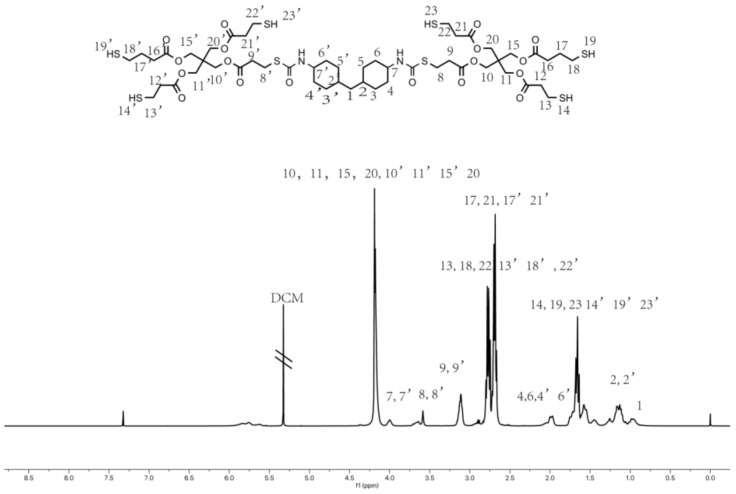
^1^H-NMR spectrum of hyperbranched thiol oligomer HMDI-6SH.

**Figure 5:**
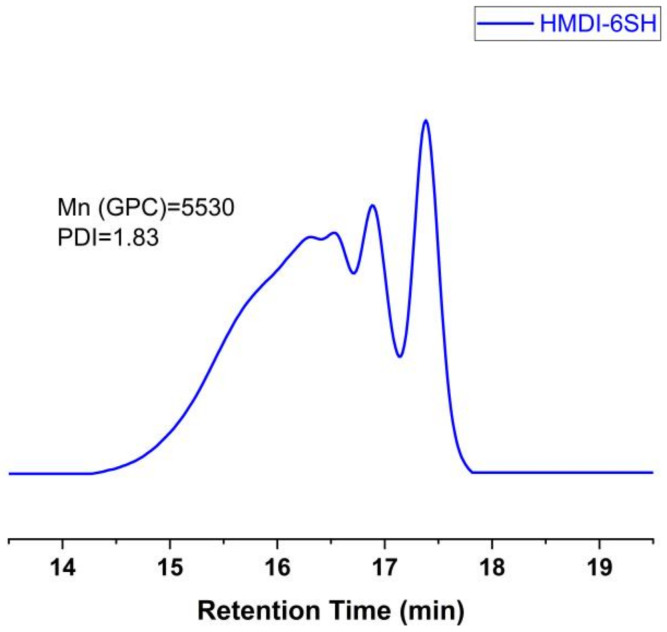
GPC traces of HMDI-6SH.

Hyperbranched oligomers do not have an accurate chemical structure with polydispersity compared to their dendritic molecules, but their synthesized process is easier than dendritic molecules without a tedious purified process. We synthesized HMDI-6SH using thiol-isocyanate click reaction via one-pot without further purification. Thiol groups can react with isocyanate groups quantitatively with Lewis bases in a dipolar solvent such as dimethyl sulfoxide (DMSO) or acetonitrile (ACN). After the reaction competition, the peak at 2230 cm^−1^ attributed to -NCO group in FT-IR spectrum was disappeared accompanied with the appearance of -SH group at 2560 cm^−1^. The chemical structure of HMDI-6SH had been confirmed by FT-IR (Figure 3) and ^1^H-NMR (Figure 4) spectrum. The average number of molecular weight (Mn) of HMDI-6SH was 5530 and PDI was 1.83 (Figure 5).

### 3.2. Degree of Conversion

**Figure 6:**
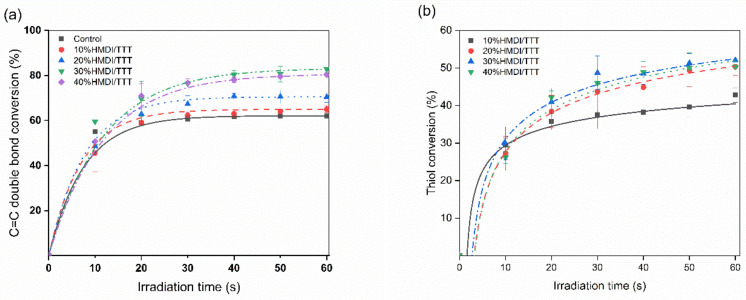
The degree of conversion of methacrylate (**a**) and thiol groups (**b**).

High final functional groups conversion for dental monomers was beneficial to the long-term material properties of dental resin systems [26]. The degree of functional groups conversion was usually monitored by real-time FTIR technology. Figure 6 showed the degree of conversion (DC%) versus irradiated time curves of methacrylate and thiol groups. It could be observed that the DC% for these two functional groups increased obviously for the first 20 s irradiation time, and the variation became slower after 20 s.

As shown in Table 1, DC% at 60 s irradiation time for methacrylate increased with the increasing of thiol-ene content in thiol-ene-methacryalte ternary resin system. A similar tendency was also observed for thiol conversion. It was indicated that HDMI/TTT thiol-ene resin system enhanced polymerization rate and significantly increased final function group conversion.

### 3.3. Volumetric Shrinkage

As seen from Table 2, after adding 10 wt% to 40 wt% of HMDI-6SH/TTT resin system into Bis-GMA/TEGDMA system, the volumetric shrinkage of dental resin was decreased from 8.52% to 4.92%. According to statistical analysis, the volumetric shrinkage of 10%HMDI/TTT systems had no statistical difference between 20%HMDI/TTT and 30% HMDI/TTT systems (*p* > 0.05), but lower than control (*p* < 0.05), and higher than 40% HMDI/TTT resin systems (*p* < 0.05).

### 3.4. Water Sorption and Water Solubility

The chemical degradation of dental resin would be accelerated by water through the oxidation process and hydrolysis [27]. Excessive water sorption and water solubility of dental resin resulted in reduced service lifetime [14]. As shown in Table 3, with the increasing amount of HDMI/TTT resin system, the water sorption of the resin was in a trend of decreasing. Compared with control resin, only 40% HMDI/TTT had higher water solubility (*p* < 0.05), all the other ternary resin systems had comparable water solubility (*p* > 0.05).

### 3.5. The Determination of Glass Transition Temperature (T_g_)

**Figure 7:**
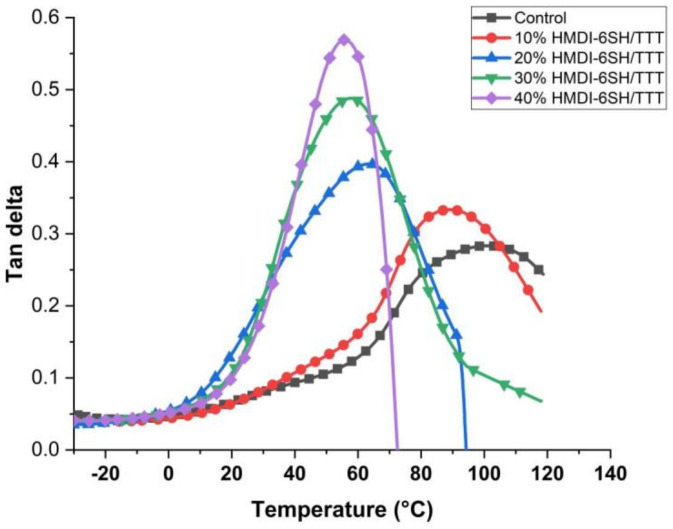
The tan δ versus temperature curves of the cured resin systems.

As shown in Figure 7, the Control group exhibited one broad peak at around 100 °C, suggesting that it was a heterogeneous polymeric network. With the concentration of HDMI-6SH/TTT thiol-ene monomers system increased, the peak of tan δ was narrowed, it was attributed to the step-growth reaction process of thiol monomers with methacrylate monomers (Bis-GMA or TEGDMA) and ene monomer (TTT) to obtain the more homogeneous network [28]. Simultaneously, the deceasing tendency of *T*_g_ was observed as given in Table 4. It was resulting from the lower crosslink density due to chain transfer reaction to thiol from ene monomer to reduce the molecular weight of the polymer and high flexible thioether moieties incorporated into the polymer network [29].

### 3.6. Flexural Properties

The results of flexural strength (FS), flexural modulus (FM), and toughness of dental resins were shown in Table 5, It was shown that FS of 10% HMDI-6SH/TTT resin and 20% HMDI-6SH/TTT resin was equivalent to that of control resin (*p* > 0.05) and higher than those of 30% HMDI/TTT resin and 40% HMDI/TTT resin (*p* < 0.05). However, when the content of HMDI-6SH/TTT monomer systems in resins was exceed 10 wt%, the FM of resin decreased from 2.52 GPa (10% HDMI/TTT) to 0.39 GPa (40% HDMI/TTT) (*p* < 0.05). It was in good agreement with several previous reports that incorporation of thiol-ene resin into methacrylate resin would decrease FS and FM [16,17,20,30].

**Figure 8:**
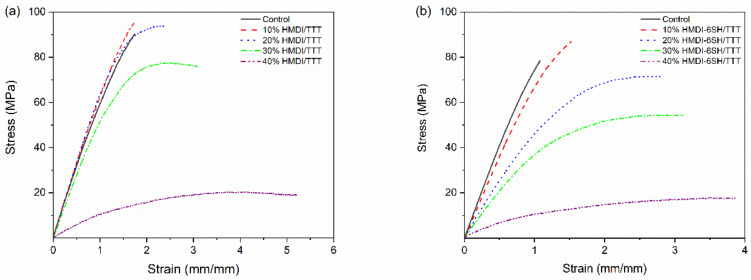
Stress/displacement curves of unfilled resins systems in three-point bending test: (**a**) before water immersion; (**b**) after water immersion.

Toughness of the resin was increased obviously after the incorporation HDMI-6SH/TTT resin (*p* < 0.05). The FS and FM of 20% HMDI-6SH/TTT resin was comparable to the Control resin (*p* > 0.05), but WF of it was about two times more than control resin (*p* < 0.05), which demonstrated that the toughness of resin was increased. Stress/displacement curves as shown in Figure 8a also indicated the remarkable increase in toughness when HDMI-6SH/TTT resin system was added into Bis-GMA/TEGDMA resin system.

With increasing the content of thiol monomers in thiol-ene-methacrylate ternary system, the molecular weight of the methacrylate oligomers decreased because of increasing the chain transfer rate for the thiol group, which could lead to the reduction of flexural modulus, hardness as well as glass transition temperatures (*T*_g_) due to the flexibility of polythioethers moieties generated from thiol-ene reaction [31,32]. As a result, the flexibility of the polymer network was increased with the increasing of polythioethers moieties, which allowed the polymer network to absorb more stress generated from flexural tests, leading to the improvement in toughness of resin [25].

## 4. Discussion

In this study, thiol groups conversion (~50%) (Table 1) was not achieved as high as expected. To our acknowledge, the thiol group conversion was mainly affected by the structure of ene monomers. Our result was in agreement with Li et al.’s research [29]. Thiol group conversion can be high as C=C double bond conversion when thiol monomer copolymerized with allyl ether monomers, divinyl ether monomers, and triallyl triazine monomers expect acrylate monomers. That’s because the thiol group conversion was suppressed by the high homopolymerization propensity of methacrylate groups.

After adding HDMI-6SH/TTT resin system into traditional Bis-GMA/TEGDMA resin, the ternary resin would have mainly three types of a chemical reaction during photopolymerization: (i) thiol-methacrylate chain transfer reaction; (ii) thiol-methacrylate and thiol-allyl triazine step-growth propagation; (iii) methacrylate-methacrylate chain propagation. The reaction rate of the thiol-methacrylate chain transfer reaction was higher than the latter two reactions, delaying the gel-point until high functional groups conversion [33]. The results of DC% in this study were consistent with previous studies on thiol-ene-methacrylate systems [14,20].

Previous studies have demonstrated that thiol-ene based resin had lower shrinkage than methacrylate-based resin [16,26]. With the increase of concentration of HMDI-6SH/TTT resin systems, the trendy of volumetric shrinkage of dental resins was decreasing. Although the conversion rate of monomers of thiol-ene-methacrylate ternary resin was higher than methacrylate-based resin, but volumetric shrinkage was still lower than methacrylate based resin, the result was in good agreement with the previous study [16]. The step-growth mechanism of thiol-ene and thiol-methacrylate polymerization led to lower volumetric shrinkage than traditional methacrylate-based resin. On the other hand, hyperbranched oligomers HMDI-6SH with a lower concentration of functional groups and lower free volume might also decrease volumetric shrinkage further [24].

The mole ratio of thiol to ene functional groups play an essential role in the physical properties of thiol-ene-methacrylate ternary resins such as flexural properties, volumetric shrinkage, and *T*_g_. At low mole ratio of thiol to ene (5~20%), flexural modulus, and *T*_g_ compare to pure methacrylate resins. Simultaneously, the polymerization rate of methacrylate was enhanced [15]. However, at a high mole ratio of thiol to ene functional groups, more chain transfer occurs throughout the network formation leading to reduced methacrylate chain length and lower crosslink density and glass transition temperature while enhancing structure homogeneity and final methacrylate groups conversion [28].

In this study, when the content of HMDI-6SH/TTT thiol-ene reached 40 wt%, the crosslink density of the resin system was reduced due to chain transfer through the network formation leading to lower crosslink density. The polymer chains become looser, and the resin changes from a glassy state to a rubbery state. The dramatical reduction of flexural strength and flexural modulus of 40%HDMI-6SH/TTT also confirmed the hypothesis (Table 5). It was the reason why the volumetric shrinkage of 40%HDMI-6SH/TTT reduced dramatically compared to other resins systems.

Several factors would influence water uptake value [34]. Among them, the hydrophilicity of the polymer matrix affected the water absorption of resin. Polar functional groups in a polymer matrix, such as hydroxyl groups, which created hydrogen bonds with water, would increase the water sorption [35]. On the contrary, Hydrophobic functional group such as thiol groups or thioether moieties reduced the water sorption of resin [36]. In the present work, there were two hydroxyl groups in one Bis-GMA molecular, so with increasing of HMDI-6SH/TTT thiol-ene system in resin, the content of hydroxyl groups in resin was reduced. At the same time, the content of thiol groups or thioether moieties in resins increased, leading to the enhancement of resin hydrophobicity. As a result, the reducing trendy of water sorption of resins was observed.

The water solubility of dental resins was affected by the type of monomers [37], DC%, the type of fillers, and fillers concentration [38]. In general, the lower DC%, the easier for residual monomers leaching out from dental resin. Although the ternary resin system had comparable or higher double bond DC% than Bis-GMA/TEGDMA based resin as shown in Table 1, their water solubility still had no significant difference, except for 40%HMDI-6SH/TTT resin system, which had the highest water solubility. It should be explained that the molecular weight of the resin was decreased with the increasing chain transfer rate due to increasing the concentration of thiol oligomer HMDI-6SH. Furthermore, the flexibility and free volume of the polymeric network were increased with increasing the content of thioethers moieties and HMDI-6SH, which led to maximation of network expansion, increasing the mobility of residual monomers or oligomers [37].

In order to evaluate the flexural properties of resin in the oral environment, the three-point bending test specimens were immersed in water at 37 °C for at least 30 days. As shown in Table 5 and Figure 8b, the decreasing trendy of flexural strength (FS), flexural modulus (FM), and toughness of the resins were observed due to the plasticization effect of water molecules after water immersion, because water molecules penetrated the polymer network [37]. The lower water sorption of the resin that reduces the plasticization effect of water in the polymer matrix was beneficial for keeping flexural properties of dental resin in the oral moist environment [14]. As shown in Table 3, the water sorption value and water solubility value of 10%HMDI-6SH/TTT resin system and 20%HMDI-6SH/TTT resin system were lower or comparable to Bis-GMA/TEGDMA control and their FS and WF shown higher or equal than Bis-GMA/TEGDMA based control after water immersion. According to the results shown in Table 5, when the content of HMDI-6SH/TTT was not more than 20 wt% in the ternary resin system, flexural properties after water immersion, water sorption, and solubility were all not influenced negatively. These results indicated that the stability of the resins was not sacrificed after adding a certain amount of HDMI/TTT monomer systems into Bis-GMA/TEGDMA based resin.

## 5. Conclusions

In Summary, a hyperbranched oligomer HMDI-6SH was synthesized via thiol-isocyanate click reaction without further purification. Its chemical structure was characterized by FT-IR and ^1^H NMR spectrum. The result showed that when the mass ratio of HDMI-6SH/TTT thiol-ene monomers in the resins was not more than 20 wt%, the higher DC%, higher toughness, lower volumetric shrinkage, lower water sorption, and lower solubility for the resins were obtained. These advantages were a benefit to improve the service life for Bis-GMA/TEGDMA based resins.

With limitation of this study, although the volumetric shrinkage of Bis-GMA/TEGDMA resin systems have been reduced by adding HMDI-6SH/TTT resin system, shrinkage stress of the resins would be investigated in future. The odor of thiol monomers should be considered in the practical applications, some other properties such as biocompatibility should be also studied in future.

## Figures and Tables

**Table 1:** The degree of conversion C=C double bond and thiol group at 60 s.

Resins	C=C Double Bond Conversion at 60 s (%)	Thiol (-SH) Conversion at 60 s (%)
Control	62.1 ± 1.1 ^a^	-
10%HMDI-6SH/TTT	63.8 ± 1.8 ^a^	42.8 ± 2.0 ^a^
20%HMDI-6SH/TTT	68.1 ± 2.0 ^b^	50.4 ± 2.3 ^b^
30%HMDI-6SH/TTT	82.8 ± 1.5 ^c^	54.1 ± 1.4 ^b^
40%HMDI-6SH/TTT	80.1 ± 1.9 ^c^	50.3 ± 1.4 ^b^

^a^ The same lower case letters indicated no statistical differences within a column (Tukey’s test, *p* = 0.05).

**Table 2:** The volumetric shrinkage of the unfilled resins.

Resins	Volumetric Shrinkage (%)
Control	8.53 ± 0.22 ^a^
10%HMDI-6SH/TTT	6.59 ± 0.25 ^b^
20%HMDI-6SH/TTT	6.72 ± 0.23 ^b^
30%HMDI-6SH/TTT	6.50 ± 0.26 ^b^
40%HMDI-6SH/TTT	4.92 ± 0.13 ^c^

^a^ The same lower case letters indicated no statistical differences within a column (Tukey’s test, *p* = 0.05).

**Table 3:** Water sorption and water solubility of unfilled resins.

Resins	Water Sorption (%)	Water Solubility (%)
Control	2.15 ± 0.09 ^a^	1.33 ± 0.03 ^a^
10%HMDI-6SH/TTT	2.05 ± 0.08 ^a,b^	1.11 ± 0.09 ^a^
20%HMDI-6SH/TTT	1.93 ± 0.04 ^b,c^	1.04 ± 0.11 ^a^
30%HMDI-6SH/TTT	1.60 ± 0.09 ^d^	1.29 ± 0.10 ^a^
40%HMDI-6SH/TTT	1.41 ± 0.12 ^d^	1.79 ± 0.12 ^b^

^a^ The same lower case letters indicated no statistical differences within a column (Tukey’s test, *p* = 0.05).

**Table 4:** The glass transition temperature (*T*_g_) of the cured resins systems determined by DMA.

Resins	*T*_g_ (°C)
Control	100
10% HMDI-6SH/TTT	89
20% HMDI-6SH/TTT	63
30% HMDI-6SH/TTT	57
40% HMDI-6SH/TTT	55

**Table 5:** The flexural strength (FS), flexural modulus (FM) and toughness of unfilled resins before and after water immersion.

Resins	Before Water Immersion			After Water Immersion		
	FS (MPa)	FM (GPa)	TS (KJ/m^2^)	FS (MPa)	FM (GPa)	TS (KJ/m^2^)
Control	92.5 ± 5.9 ^a,A^	2.53 ± 0.23 ^a,A^	4.76 ± 0.92 ^a,A^	76.7 ± 8.7 ^a, B^	2.44 ± 0.02 ^a,A^	3.40 ± 0.41 ^a,B^
10%HMDI-6SH/TTT	93.4 ± 4.0 ^a,A^	2.52 ± 0.25 ^a,A^	5.46 ± 0.31 ^a,A^	88.8 ± 2.9 ^b,A^	2.54 ± 0.16 ^a,A^	5.07 ± 0.59 ^b,A^
20%HMD-6SH/TTT	91.4 ± 2.0 ^a,A^	2.20 ± 0.05 ^b,A^	9.75 ± 0.97 ^b,A^	76.0 ± 5.3 ^a,B^	1.90 ± 0.25 ^b, B^	8.09 ± 1.32 ^c,A^
30%HMDI-6SH/TTT	79.3 ± 5.9 ^b,A^	1.95 ± 0.18 ^c,A^	10.52 ± 1.93 ^b,A^	58.9 ± 3.5 ^c,B^	1.35 ± 0.13 ^c,B^	7.96 ± 1.68 ^c,A^
40%HMDI-6SH/TTT	21.4 ± 1.2 ^c,A^	0.39 ± 0.06 ^d,A^	8.22 ± 1.29 ^b,A^	17.7 ± 4.0 ^d,A^	0.26 ± 0.09 ^d,A^	5.90 ± 1.24 ^b,B^

^a^ The same lower case letters indicated no statistical differences within a column (Tukey’s test, *p* = 0.05). ^A^ The same upper case letters indicated no statistical differences between the same property before and after water immersion (Tukey’s test, *p* = 0.05).

## Data Availability

The data presented in this study are available on request from the corresponding author. The data are not publicly available due to IPR considerations.
